# Unexpected Intramedullary Canal Fill During Ultrasound-Guided Subchondral Intraosseous Platelet-Rich Plasma Injection: A Case Report

**DOI:** 10.7759/cureus.92187

**Published:** 2025-09-12

**Authors:** Tsung Ju Wu, Wei-Cheng Liao, Chih-Wei Lee

**Affiliations:** 1 Regenerative Medicine, Reboot Clinics, Changhua, TWN; 2 Physical Medicine and Rehabilitation, Changhua Christian Hospital, Changhua, TWN; 3 Pain Management, Prolocare Clinic, Taichung, TWN; 4 Radiology, Changhua Christian Hospital, Changhua, TWN

**Keywords:** bone marrow lesion, intraosseous, osteoarthritis, platelet-rich plasma, subchondral, ultrasound

## Abstract

Ultrasound (US)-guided subchondral intraosseous (IO) injection is increasingly utilized for treating knee osteoarthritis (OA) associated with bone marrow lesions (BMLs). However, accidental intramedullary misplacement ("canal fill") during this procedure is a rarely documented complication. We report an 83-year-old female patient with painful knee OA and medial compartment BML who underwent US-guided subchondral IO platelet-rich plasma (PRP) injection. Due to osteoporosis, the initial needle insertion inadvertently penetrated deeper into the intramedullary canal of the tibia. Fluoroscopy revealed intramedullary contrast medium spread, correlating with a compact, ball-shaped Doppler hotspot under US Power Doppler Imaging (PDI). Needle repositioning towards the subchondral bone resulted in a typical thin, sheet-like Doppler blush pattern beneath the cortical bone. Subsequent PRP IO injections provided significant symptomatic relief after one month, reducing pain from a visual analog scale (VAS) score of 7 to 2. Recognizing US features indicative of intramedullary IO misplacement is crucial. Real-time US imaging, specifically observing Doppler distribution patterns, can prevent inadvertent intramedullary injections, ensuring precise subchondral delivery and optimal therapeutic outcomes.

## Introduction

Osteoarthritis (OA) of the knee is one of the leading causes of mobility loss and disability among older adults worldwide, contributing to chronic pain and impaired quality of life [1]. As the disease progresses, degenerative changes involve the subchondral bone, hyaline cartilage, synovium, menisci, and ligaments, and late-stage OA is frequently accompanied by varus or valgus axial deformity [2]. Most patients with end-stage (Kellgren and Lawrence grade 3-4) knee osteoarthritis who have failed non-operative therapy ultimately undergo total knee arthroplasty [3]. Knee OA pain is driven by synovitis, subchondral bone marrow lesions (BMLs), and effusion, each showing measurable associations with symptoms [4]. The prevalence of symptomatic knee OA continues to rise, driven largely by global population ageing and the worldwide surge in obesity [1].

Subchondral intraosseous (IO) orthobiologic injection attracted more attention in recent years. In a controlled study of 48 rats with monosodium iodoacetate (MIA)-induced knee osteoarthritis, combining subchondral IO and intra-articular (IA) injections of platelet-rich plasma (PRP) or bone marrow aspirate concentrate (BMAC) yielded stronger, longer-lasting pain relief, attenuated cartilage degeneration, and better preserved subchondral bone than IA injection alone [5]. In an observational cohort of 60 patients with Ahlbäck grade III-IV knee osteoarthritis, adding subchondral IO PRP infiltrations to the IA PRP regimen led to statistically and clinically significant improvements across all Knee Injury and Osteoarthritis Outcome Score (KOOS) and Western Ontario and McMaster Universities Arthritis Index (WOMAC) domains at six and 12 months [6]. In a 15-year randomized study of 60 patients with bilateral knee osteoarthritis, subchondral implantation of bone marrow-derived mesenchymal stem cell concentrate postponed total knee arthroplasty far more effectively than injecting the same dose intra-articularly into the contralateral knee (total knee arthroplasty incidence 1.3% vs 4.6% per knee-year; 20% vs 70% knees converted) and also achieved superior clinical and magnetic resonance imaging (MRI) improvements at two years [7].

Traditionally subchondral IO orthobiologic injection is performed under fluoroscopy guidance, and a 13-gauge (G) trocar is advanced into the subchondral area [8]. In the knee joint, the trocar is aimed towards the joint line to reach the subchondral area. This procedure requires sedation to reduce patients’ discomfort. Recently an ultrasound (US)-guided subchondral IO approach has been proposed [9]. Instead of a trocar and subsequent need of sedation, a 21-G needle is placed under local anesthetics to penetrate the cortex. US-guided procedures could be performed in a clinic setting and free of radiation. The flow of injectae can be observed under power Doppler image (PDI) mode. However, the accuracy of US guidance for subchondral IO injections, compared with fluoroscopy, has not yet been established. In this case report, we report an unexpected intramedullary canal fill during US-guided subchondral PRP injection. Also, we observe the different characteristics of images under US between subchondral IO and intramedullary IO injection.

## Case presentation

**Figure 1:**
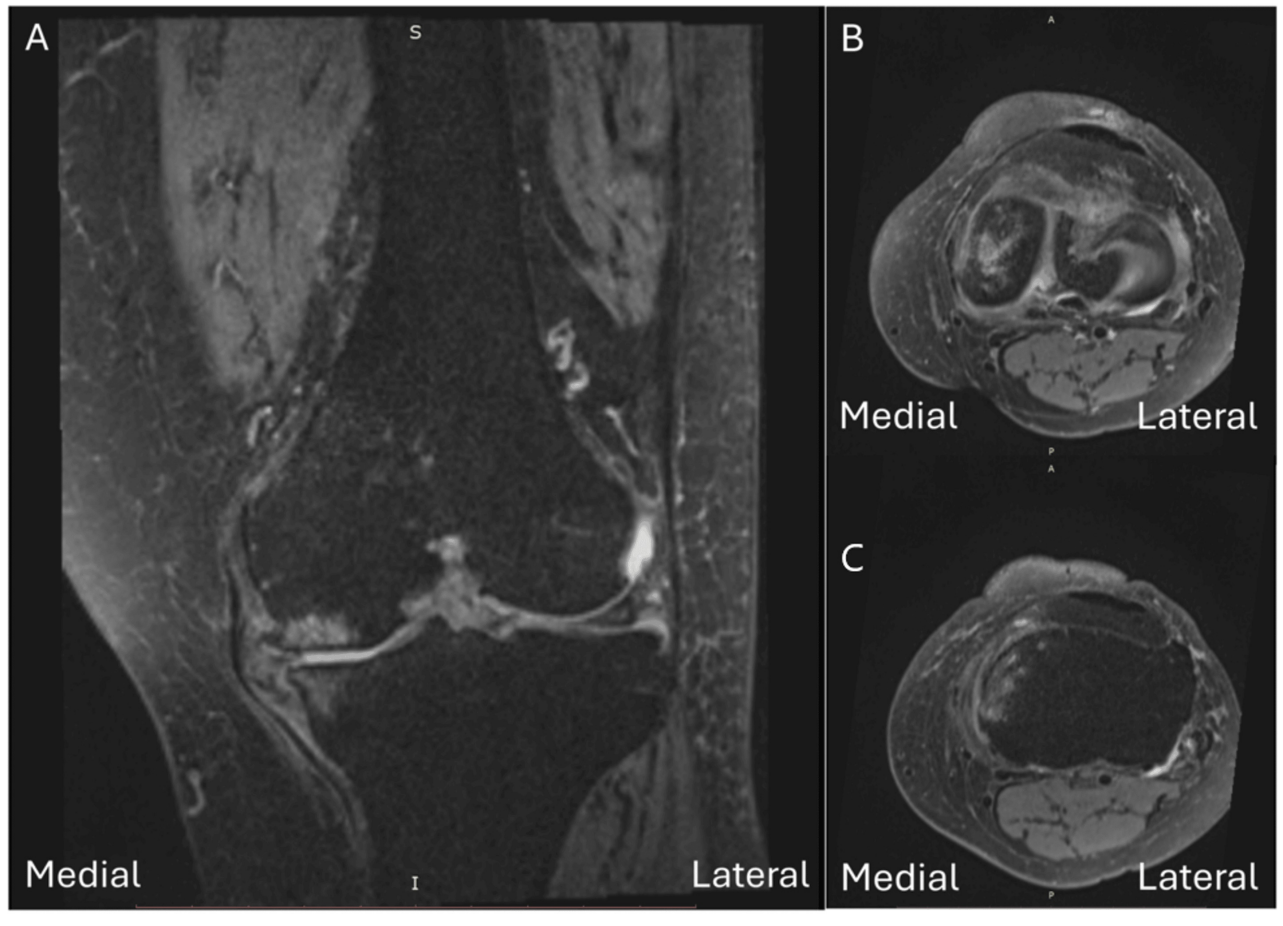
Magnetic resonance imaging (MRI) findings on proton density–weighted fat-suppressed fast spin echo (PDw FS FSE) sequences. (A) Coronal image shows high signal intensity in the medial femoral condyle and medial tibial plateau, consistent with bone marrow lesions, together with a grade 3 medial meniscus tear with extrusion. (B) Axial image shows high signal intensity in the medial femoral condyle. (C) Axial image shows high signal intensity in the medial tibial plateau.

This 83-year-old female patient presented with left painful knee joint. Her discomfort was located medially for two years. The visual analog score (VAS) of her knee pain was 7 in her daily life. X-ray revealed grade III osteoarthritis change according to Kellgren and Lawrence classification. MRI revealed medial meniscal protrusion with grade 3 tear. BML was noted over the medial femoral condyle and tibial plateau (Figure 1).

**Figure 2:**
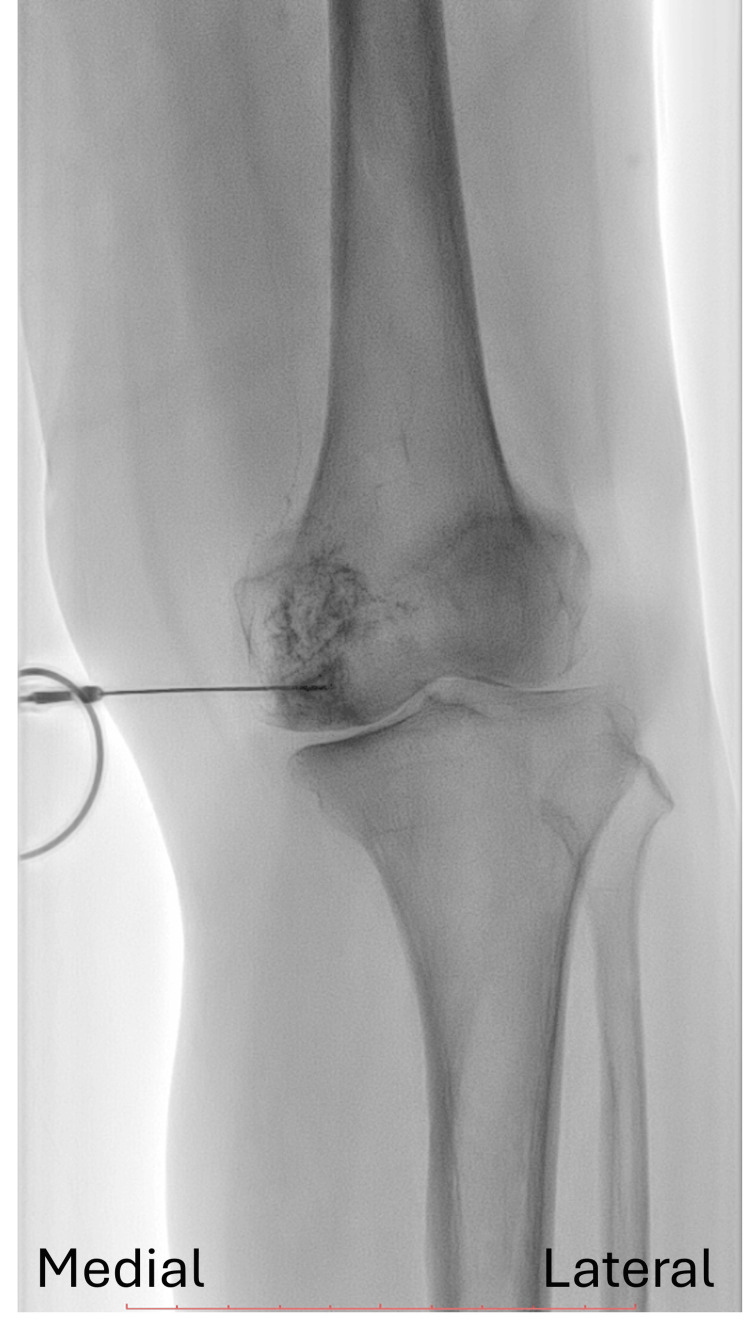
Fluoroscopic imaging demonstrated a cloud-like distribution of contrast medium confined within the medial femoral condyle, consistent with subchondral intraosseous (IO) injection.

Her symptoms did not improve after arthrocentesis and intra-articular hyaluronic acid injection. After discussion with the patient, subchondral IO PRP injection was arranged in the fluoroscopy room. RegenKit®-THT® (Regen Lab, Lausanne, Switzerland) tube was used to prepare PRP. We also use US to guide the trajectory of needle insertion. After skin numbing with local anesthetics (1% Lidocaine), a 21-G, 2 3/4-inch needle was inserted under US guidance using out-of-plane technique with linear probe. The medial femoral condyle IO injection was performed first. The needle trajectory was oriented perpendicular to the cortical surface of the medial femoral condyle. Under fluoroscopy, the contrast medium (iobitridol 350 mg/mL; Xenetix, Guerbet, France) demonstrated a cloud-like distribution confined to the medial femoral condyle, consistent with subchondral injection (Figure 2).

**Figure 3:**
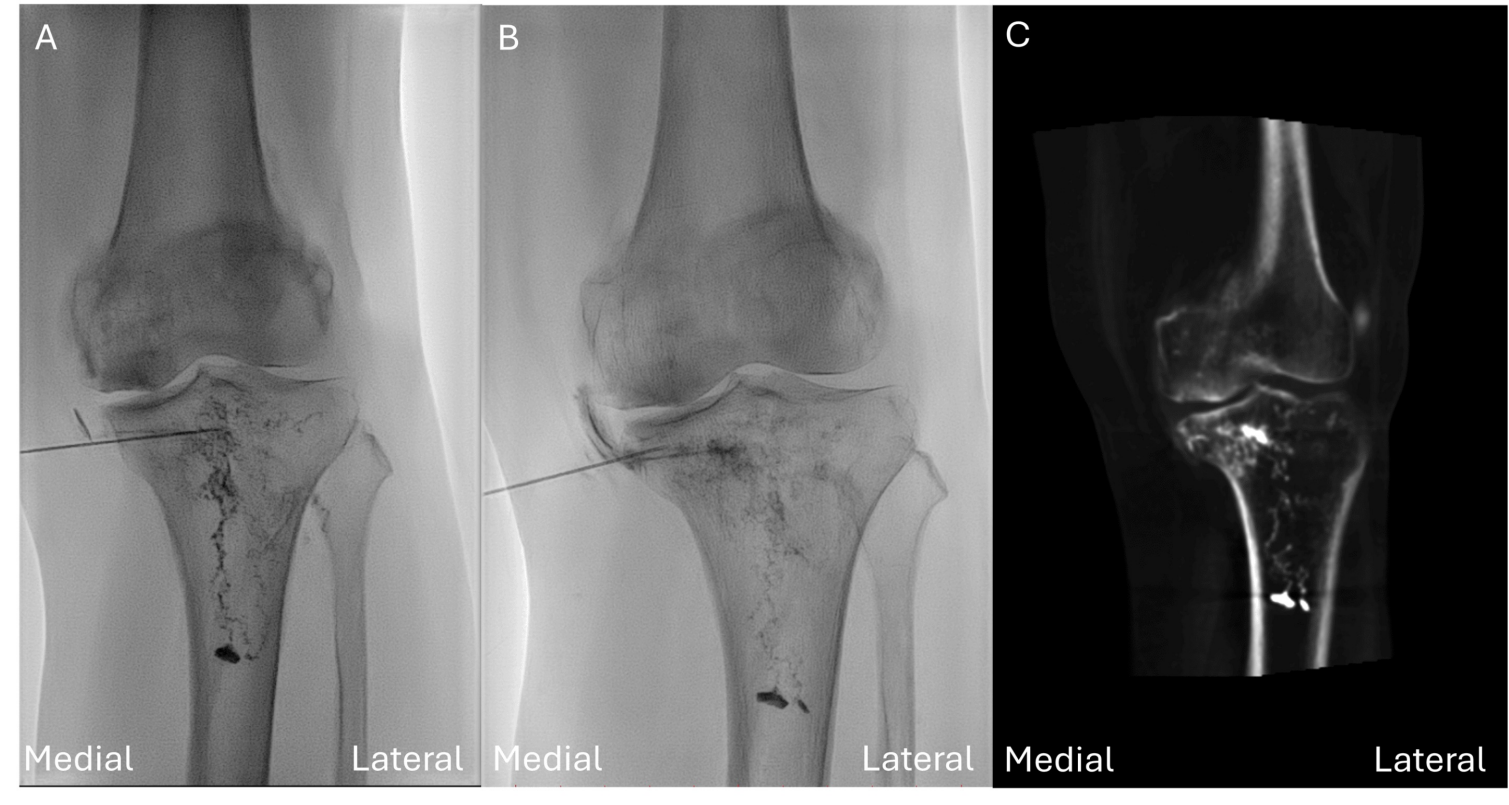
(A) Fluoroscopy showing a characteristic intramedullary intraosseous (IO) contrast medium pattern. (B) After needle withdrawal and repositioning towards the joint line, fluoroscopy demonstrated a subchondral contrast pattern. (C) Cone beam computed tomography (CBCT) confirming the subchondral spread pattern.

Subsequently, 5 mL of PRP was injected intraosseously into the subchondral area. However, the first attempt of the medical tibial plateau IO injection was deeper than we expected due to osteoporosis of this elderly patient. Fluoroscopy revealed a characteristic intramedullary IO contrast medium pattern (Figure 3A).

**Figure 4:**
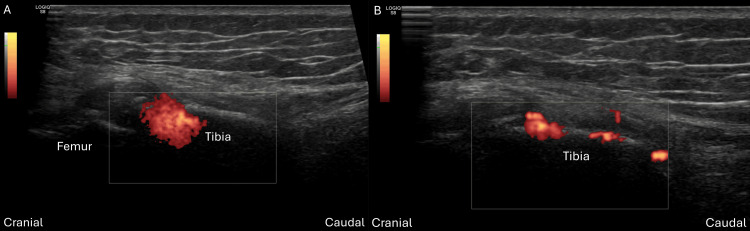
(A) The ultrasound (US) image in power Doppler imaging (PDI) mode showed a compact, ball-shaped Doppler hotspot. (B) After needle repositioning, the subsequent US image in PDI mode demonstrated a thin, sheet-like Doppler blush spreading evenly along the undersurface of the cortical line.

Then US image in PDI mode revealed a compact, ball-shaped Doppler hotspot (Figure 4A), tightly confined to the needle tip with no diffuse extension into the surrounding cortex.

After we withdrew and repositioned the needle towards the joint line, we obtained a subchondral image under fluoroscopy (Figure 3B). The following US image in PDI mode revealed a thin, sheet-like Doppler blush that spread evenly along the undersurface of the cortical line (Figure 4B). Further cone beam computed tomography (CBCT) confirmed the subchondral spread pattern (Figure 3C). Then 5 ml PRP was injected into the tibial plateau. One month later, the patient reported significant symptom relief and her VAS score dropped from 7 to 2.

## Discussion

BML, detected under MRI, is suggestive of the presence of subchondral bone damage. BML is found to be associated with a wide range of pathological conditions, such as trauma, osteoarthritis, insufficiency fracture, and osteonecrosis [10]. In symptomatic knee OA patients, the prevalence of BML is higher than in asymptomatic knee OA patients [11]. BML is unlikely to resolve and often gets larger over time if left untreated, and the enlargement of BML is commonly observed in patients with deterioration of knee OA from K-L grade 2 to K-L grade 3 in long-term follow-up [12]. Furthermore, enlarging or new BML occurred mostly in malaligned limbs, on the side of the malalignment [13]. For example, medial femoral condyle and tibial plateau BMLs could be observed in varus deformity patients. Subchondral IO injection is a common procedure to ameliorate patients’ symptoms. Calcium phosphate (CaP), PRP, and BMAC were commonly used regimens [14]. Long-term studies show that treating the subchondral bone instead of intra-articular injection lowers the likelihood of total knee replacement, eases clinical symptoms, and produces measurable MRI improvements [7]. It is a treatment option for patients with painful knee osteoarthritis accompanied by BMLs.

In this case report, we accidentally inserted the needle deeper than we expected into the intramedullary IO canal due to osteoporosis of the patient. Intramedullary IO access is a rapid vascular access technique commonly used in emergent and critical care settings [15]. When traditional intravenous access is difficult or time-consuming, intramedullary IO allows direct insertion of a needle into the medullary cavity of long bones. The rich non-collapsible venous plexus within the bone marrow enables quick administration and absorption of medications, fluids, and blood products into the central circulation. Under fluoroscopy, the spread pattern of contrast medium differs between subchondral and intramedullary IO injections. Subchondral IO injections typically produce a localized, cloud-like distribution confined immediately beneath the cortical bone, reflecting limited permeability and higher resistance within subchondral trabecular bone [16]. In contrast, intramedullary IO injections generate a linear or tubular pattern with the contrast agent rapidly disseminating along the marrow cavity, indicating lower resistance and greater vascularity within the medullary space. We observed distinct injectate distribution patterns under US: subchondral IO injections produced a thin, sheet-like, homogeneous Doppler blush evenly distributed beneath the cortical surface, whereas intramedullary IO injections showed a compact, ball-shaped Doppler hotspot confined closely around the needle tip. Our needle trajectory differed from prior reports. Fluoroscopy-guided techniques typically recommend an insertion angle of approximately 45° toward the joint line [17]. In contrast, under US guidance we introduced the needle perpendicular to the cortical surface. Compared with the prior study, our approach aims more horizontally relative to the joint surface and slightly off the joint line, which may reduce the risk of intra-articular breach. Precise control of needle depth is essential to prevent intramedullary infiltration (canal fill). Therefore, higher resistance with back pressure and sheet-like homogeneous Doppler blush pattern under US indicated successful subchondral IO injection. Furthermore, pre-filling the subchondral space with local anesthetics before IO injection could ameliorate the discomfort of the whole procedure.

## Conclusions

This case highlights the importance of real-time US imaging and the ability to recognize distinct Doppler signal patterns in differentiating intramedullary versus subchondral IO injections. If the injectate enters the intramedullary space, it will not remain within the subchondral compartment, thereby leading to ineffective treatment. Immediate identification of a compact, ball-shaped Doppler hotspot together with a sudden loss of back pressure (low injection resistance) should be considered a sign of intramedullary malposition and should prompt immediate needle repositioning towards the subchondral zone. Accurate interpretation of US findings significantly enhances procedural safety, efficacy, and patient outcomes in US-guided subchondral IO orthobiologic injections. To our knowledge, this is the first article reporting the US features of inadvertent intramedullary IO misplacement during subchondral PRP IO injections. Furthermore, canal-fill-type intramedullary spread in a verified subchondral needle position has not been reported. In this case it was observed only when the needle tip had advanced into the medullary cavity. Future large-scale studies are warranted to estimate the incidence of intramedullary canal fill during subchondral-targeted IO injections, ideally with fluoroscopic or CBCT confirmation.
